# Methylation Levels in the Promoter Region of *FHIT* and *PIAS1* Genes Associated with Mastitis Resistance in Xinjiang Brown Cattle

**DOI:** 10.3390/genes14061189

**Published:** 2023-05-29

**Authors:** Liwei Zhong, Shengchao Ma, Dan Wang, Menghua Zhang, Yuezhen Tian, Junmin He, Xiaoxue Zhang, Lei Xu, Cuiling Wu, Mingming Dong, Murong Gou, Xixia Huang, Kechuan Tian

**Affiliations:** 1 College of Animal Science, Xinjiang Agricultural University, Urumqi 830091, China; zhongliwei0605@163.com (L.Z.); shengchaomasicau@163.com (S.M.); wangdan01100330@163.com (D.W.); zhangmenghua810@126.com (M.Z.); zhangxiaoxue0726@163.com (X.Z.); q609468041@sina.com (L.X.); cuiling_wu@163.com (C.W.); dongmingming@xdmy.com (M.D.); goumurong_2162@163.com (M.G.); 2 Quality Standards Institute of Animal Husbandry, Xinjiang Academy of Animal Sciences, Urumqi 830011, China; 3 Key Laboratory of Genetics Breeding and Reproduction of Xinjiang Wool Sheep and Cashmere-Goat, Institute of Animal Science, Xinjiang Academy of Animal Sciences, Urumqi 830011, China; tian306656637@126.com; 4 Institute of Animal Science and Veterinary Medicine, Shandong Academy of Agricultural Sciences, Jinan 250100, China; hejunmin330@163.com

**Keywords:** Xinjiang brown cattle, mastitis resistance, GWAS, DNA methylation, candidate genes, *FHIT* and *PIAS1* genes

## Abstract

Mastitis causes serious economic losses in the dairy industry, but there are no effective treatments or preventive measures. In this study, the *ZRANB3*, *PIAS1*, *ACTR3*, *LPCAT2*, *MGAT5*, and *SLC37A2* genes in Xinjiang brown cattle, which are associated with mastitis resistance, were identified using a GWAS. Pyrosequencing analysis showed that the promoter methylation levels of the *FHIT* and *PIAS1* genes in the mastitis group were higher and lower, respectively, than those in the healthy group (65.97 ± 19.82% and 58.00 ± 23.52%). However, the methylation level of the *PIAS1* gene promoter region in the mastitis group was lower than that in the healthy group (11.48 ± 4.12% and 12.17 ± 4.25%). Meanwhile, the methylation levels of CpG3, CpG5, CpG8, and CpG15 in the promoter region of the *FHIT* and *PIAS1* genes in the mastitis group were significantly higher than those in the healthy group (*p* < 0.01), respectively. RT-qPCR showed that the expression levels of the *FHIT* and *PIAS1* genes were significantly higher in the healthy group than those in the mastitis group (*p* < 0.01). Correlation analysis showed that the promoter methylation level of the *FHIT* gene was negatively correlated with its expression. Hence, increased methylation in the promoter of the *FHIT* gene reduces the mastitis resistance in Xinjiang brown cattle. Finally, this study provides a reference for the molecular-marker-assisted selection of mastitis resistance in dairy cattle.

## 1. Background

Mastitis is one of the most common diseases in dairy cattle and an important factor hindering the development of the modern dairy industry. Mastitis not only affects the milk yield and quality, health, and other aspects of dairy cattle, but also can lead to premature elimination of dairy cattle [1]. However, there are no effective treatments or preventive measures for dairy cattle mastitis. Studying the molecular regulation mechanism of mastitis and mastitis resistance in cattle breeds can provide a reference for the prevention and treatment of mastitis [2,3,4,5,6].

Among the many molecular mechanisms, DNA methylation is critical to genome structure stability and an important way to regulate gene expression, associated with increased/decreased productivity and prevalence rates in some animals [7,8,9]. Previous studies have found that DNA methylation regulates the expression of multiple genes during mammary gland development and further, specifically affects mammary epithelial cell proliferation, survival, and differentiation, the formation of terminal milk buds, duct lengthening, lobular alveoli, and fat development [10,11,12]. For example, Wang et al. compared the sequence methylation and expression level of the *CDH13* gene between Chinese Holstein cows with mastitis and healthy cows, and the results showed that there was no significant difference in methylation of *CDH13* between the two groups, but the gene expression was up-regulated in the mastitis group, indicating that this may affect the occurrence of mastitis [10]. Zhang et al. investigated the DNA methylation of the *IL6R* gene exon 2 in the mammary gland tissue of cattle with confirmed mastitis, and they found that the gene was hypermethylated and its expression was affected. The results suggested that the *IL6R* gene may regulate cattle mastitis by modifying DNA methylation modification [11]. Wang et al. compared the sequence methylation levels of the *TRAPPC9* and *CD4* genes between a mastitis group and a healthy group of Xinjiang brown cattle, and the results showed that the sequence methylation level of *TRAPPC9* was higher and *CD4* genes was lower in the mastitis group than in the healthy group [13]. Similar results were obtained in a study by Zhang et al. [14]. In conclusion, it is indisputable that DNA methylation partly affects the regulation of mammary gland development; however, the complex relationships among the different genes it affects remain to be further studied [15].

Xinjiang brown cattle (*Bos taurus*) are valuable for both meat and milk production in Xinjiang, China. Xinjiang brown cattle were created by crossing local cattle as the mother, Swiss brown cattle, Aratoma, and a few Costrom cattle as the father, and were then selected for a long time. Xinjiang brown cattle have strong adaptability, cold tolerance, and extensive feeding, strong disease resistance, and high milk and meat production performance. The maximum milk yield of Xinjiang brown cattle (milk type) reached 7650 kg/305 days under house feeding conditions, while the milk yield of Xinjiang brown cattle (dairy-type) was generally 1742.8–3419.6 kg/150 days under grazing conditions. Xinjiang brown cattle are distributed in all the grazing areas of Xinjiang, with the central producing areas in Yili and Tacheng. Meanwhile, Xinjiang brown cattle (mean somatic cell number: 39.24 ± 60.41 × 104/mL) have desirable characteristics compared to Holstein cattle (mean somatic cell number: 61.15 ± 89.2 × 104/mL), such as low somatic cell count and high milk fat and protein percentage. (Our group obtained these data by analyzing the DHI records of 2904 Holstein cattle and 468 Xinjiang brown cattle over 16 months (2006–2009) in Xinjiang) Therefore, Xinjiang brown cattle is an ideal material to study cow mastitis resistance However, the molecular disease resistance breeding of Xinjiang brown cattle is relatively lagging among cattle breeds, and there is still a large amount of genetic information related to the regulation of mastitis in the genome of Xinjiang brown cattle that has not been fully revealed.

In this study, Xinjiang brown cattle were used as the research object, and the 150k SNP dataset and milk somatic cell counts of 403 Xinjiang brown cattle were used for GWAS in order to screen the candidate genes associated with mastitis resistance. In addition, we further studied the impact of promoter sequence methylation levels on the expression of two candidate genes (*FHIT* and *PIAS1* genes) by using pyrosequencing, RT-qPCR, etc. (The *FHIT* gene as a candidate gene associated with mastitis resistance was screened in Xinjiang brown cattle based on our previous research [16]). Our research provides a new reference for improving mastitis resistance in dairy cattle breeds.

## 2. Materials and Methods

### 2.1. Sample Collection

In this study, 12 Xinjiang brown cattle (dairy-type) in the same feeding conditions were selected from the brown cattle breeding center of the local state-run Urumqi cattle breeding farm. These 12 animals were selected based on DHI records, with ages over 6 months, latent mastitis test records, and veterinary diagnoses. Then, based on the recommended classification standard of somatic cell count (SCC) in milk formulated by the International Dairy Federation (IDF), the 12 Xinjiang brown cattle were further divided into 2 groups: a control group (*n* = 6, SCC ≤ 200,000/mL) and a mastitis group (*n* = 6, SCC ≥ 1,000,000/mL).

### 2.2. DNA and RNA Extraction

The genomic DNA in the blood of Xinjiang brown cattle was extracted using the phenol–chloroform method [17]. The following are the steps for extracting DNA: (1) The 800 µL blood was placed in a 2 mL Ep tube, and 1000 µL T_10_E_10_ (reagent preparation method: 500 mL T_10_E_10_ contained 0.605 g Tris and 1.8612 g EDTA) was added to this 2 mL Ep tube. The Ep tube was shaken for 10 min to break up the red blood cells and then low-temperature centrifuged at 8000 r/min for 6 min to precipitate the white blood cells. (2) The upper liquid was removed; then, 800 µL T_10_E_1_ was added (reagent preparation method: 500 mL T_10_E_10_ contained 0.605 g Tris and 0.18612 g EDTA) to the Ep tube, the Ep tube was shaken for 5 min, and then it was low-temperature centrifuged at 8000 r/min for 6 min. (3) The upper liquid was removed, 500 µL USSTE (500 mL USSTE contained 29.25 g Nacl, 0.605 g Tris, 4.653 g EDTA-Na·2H_2_O, 2.5 g SDS, and 0.24 g urea) was added to the Ep tube, and the pipette tip was used to blow the precipitate 4–5 times, and the Ep tube was shaken for 5 min to rupture the white blood cells. (4) A total of 500 µL Tris equilibrium phenol was added to the Ep tube, and the Ep tube was shaken for 7 min and then centrifuged at 10,000 r/min for 10 min. (5) A total of 500 µL upper liquid was absorbed and transferred to a new 1.5 mL Ep tube, 500 µL mixture of chloroform and isoamyl alcohol was added, and the Ep tube was shaken for 5 min and then centrifuged for 10 min at 10,000 r/min. (6) A total of 300 µL upper liquid was absorbed and transferred to a new 1.5 mL Ep tube. A total of 600 µL anhydrous ethanol was added to the new Ep tube, and the Ep tube was shaken for 1 min and centrifuged for 10 min at 12,000 r/min. (7) The upper liquid was removed, 500 µL 70% ethanol was added to the Ep tube, and the Ep tube was shaken for 2 mi and then centrifuged for 10 min at 12,000 r/min. (8) Step 7 was repeated. (9) The upper liquid was removed, the Ep tube was dried at room temperature for 30–40 min, 60 µLT_10_E_1_ was added, the Ep tube was shaken and left overnight at room temperature, and finally, the DNA sample was stored at −4 °C.

Total blood RNA was extracted using an RNAprep pure Blood kit (Tiangen, Beijing, China), following the kit instructions. After extraction, all DNA and RNA samples were quality controlled using agarose gel electrophoresis and a NanoDrop 2000. The RNA/DNA sample quality control standard was OD260/280 ≈ 1.8–2.0 and OD260/230 > 2.0.

### 2.3. GWAS

The original data for GWAS were the Illumina 150K SNP dataset and the SCC dataset of 403 Xinjiang brown cattle obtained in our previous study [16]. Mastitis in dairy cattle is primarily assessed by somatic cell score (SCS), which needs to be converted using somatic cell count (SCC). The conversion formula is as follows:

$$\mathrm{SCS}=\log_{2}\frac{\mathrm{SCC}}{100,000}+3$$

Plink software was used for quality control of the 150K SNP dataset [18]. The quality control standards were as follows: (1) detection rate of a single SNP site was greater than 90%, (2) genotyping rate was greater than 90%, (3) Hardy–Weinberg equilibrium *p*-value was greater than 1 × 10^−5^, (4) secondary allele frequency (MAF) > 0.05, and (5) SNPs had unknown physical location or were located on the sex chromosome. After quality control, a total of 399 Xinjiang brown cattle and 118,811 high-quality SNPs were used for GWAS analysis, and the threshold of genome-wide significance level was 4.21 × 10^−7^. FarmCPU software was further used for GWAS [19], and the SCS was used as the input data for the GWAS. FarmCPU uses fixed effects and random effects models for alternating cycle calculation. The fixed effect model is as follows:

$$Y_{i}=M_{i1}b_{1}+M_{i2}b_{2}+\ldots +M_{\mathrm{it}}b_{t}+S_{\mathrm{ij}}b_{j}+e_{i}$$

where Y_i_ is the observed value of the trait; $M_{i1}b_{1}$, $M_{i2}b_{2}$, $M_{\mathrm{it}}b_{t}$ are the significant sites in the random effects model, which are empty at first; S_ij_ is the covariable coefficient; b_j_ is the SNP genotype (0,1,2); and e_i_ is the random residual.

The random effect model is as follows:

$$Y_{i}=u_{i}+e_{i}$$

where Y is the observed value of the trait, u_i_ is the significant random effect of SNPs in the first step, and e_i_ is the random residual.

This method combines the benefits of a mixed linear model and stepwise regression, testing one SNP at a time using the iterative method. In the calculation process, more significant SNPs were obtained through the fixed effect model, but there were more false-positive results. The second step was to construct the relationship matrix of significant SNPs to reduce the false-positive results and ensure the accuracy and reliability of the results.

Then, R software was used to draw a quantile–quantile (Q–Q) plot, and the Q–Q plot was used in the group stratification test. Based on the bovine reference genome released by NCBI (*Bos taurus* UMD 3.1.1, GCA_000003055.5), the significant SNPs were annotated, and the physical location of SNPs on the reference genome was used to infer which gene the SNPs were in or near. A preliminary analysis of the biological functions of candidate genes was conducted by searching the NCBI (https://www.ncbi.nlm.nih.gov/ (accessed on 15 August 2021)), Ensembl (http://asia.ensembl.org/index.html (accessed on 15 August 2021)), and GeneCards (https://www.genecards.org/ (accessed on 15 August 2021)) databases combined with reviewing previous studies.

### 2.4. CpG Island Prediction and Primer Design in Gene Promoter Region

First, online MethPrimer software (http://www.urogene.org/methprimer/ (accessed on 15 August 2021)) [20] was used to predict the CpG islands in promoter regions of *FHIT* (GenBank: NC_037349.1) and *PIAS1* (GenBank: NC_037337.1) genes (*FHIT* promoter region: 1416–1543 bp; *PIAS1* promoter region: 1789–1972 bp) Then, PyroMark Assay Design 2.0 was used to design the pyrosequencing primers in the promoter regions of the two genes. Finally, the primers were synthesized by Saiao Biotechnology Co., Ltd. (Shanghai, China). The detailed information of pyrosequencing primers is given in Table S1.

### 2.5. Pyrosequencing

The 12 DNA samples were methylated using an EpiTect Bisulfite Kit (Qiagen, Dusseldorf, Germany). The treated DNA was stored in the refrigerator at −80 °C for further analysis. Then, PCR was performed, and the reaction system of PCR is shown in Table S2 and the amplified conditions are shown in Table S3. Finally, the PCR products were used for pyrosequencing, which was completed on the PyroMark Q48 platform at Saiao Biotechnology Co., Ltd. (Qingdao, China).

### 2.6. Quantitative Real-Time PCR

After total RNA quality control, total RNA was reverse-transcribed into cDNA by using an All-In-One 5× RT MasterMix kit (ABM, Richmond, BC, Canada), and experiments were carried out according to the kit instructions. The RT-qPCR primers of *GAPDH*, *FHIT,* and *PIAS1* genes were designed using Primer Premier 5.0 software, and the primers were synthesized by Shengong Bioengineering Co., Ltd. (Shanghai, China) The detailed information of RT-qPCR primers is given in Table S4.

Then, RT-qPCR was performed using a TB Green Premix Ex TaqⅡkit (Takara, Shanghai, China); the reaction system of RT-qPCR is shown in Table S5 and the amplified conditions are shown in Table S3.

Finally, the 2^−∆∆Ct^ method was used to calculate relative gene expression, using the following formula:

ΔCt = Target gene mean Ct − reference gene mean Ct

ΔCt_(mean of control)_ = Target gene mean Ct _(Control)_ − Reference gene mean Ct _(Control)_

ΔΔCt = ΔCt _(Experimental)_ − ΔCt _(Control)_

Relative expression = 2^−ΔΔCt^

### 2.7. Statistical Methods

In this study, Excel 2019, SPSS 19.0, and GraphPad 5 were used for statistical analysis and plotting; *p* < 0.05 was considered significant, and *p* < 0.01 was considered extremely significant.

## 3. Results

### 3.1. Quality Control of DNA Samples

**Figure 1:**
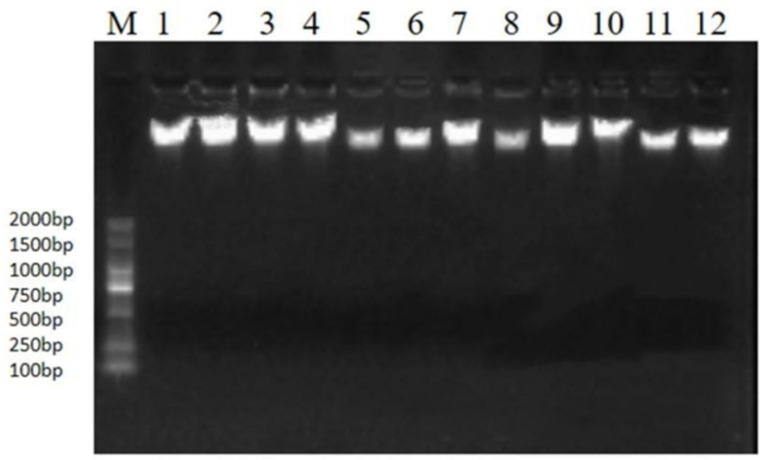
The agarose gel electrophoresis map of 12 DNA samples. M is DL2000-Marker, and No. 1–12 are 12 DNA samples.

After DNA extraction, a DNA quality test was performed; the results show that the OD (260/280) value of 12 samples was between 1.6 and 1.8, and no heterobands or other contamination were found in the samples (Figure 1), suggesting that the sample quality was good and they could be used for subsequent experiments.

### 3.2. GWAS of Mastitis Resistance Traits in Xinjiang Brown Cattle

**Figure 2:**
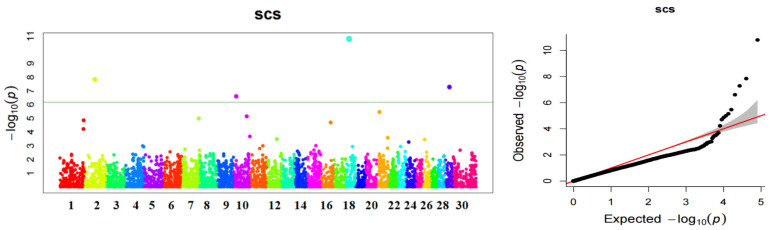
Manhattan plot and Q–Q plot of GWAS for somatic cell score of Xinjiang brown cattle mastitis. Color differences in the scatter distinguish SNPS on distinct chromosomes.

The GWAS results showed that four SNPs, located on chromosomes 2, 10, 18, and 29, were significantly associated with the somatic cell count score (Figure 2 and Table 1), and six candidate genes associated with mastitis traits were annotated (Table 1).

### 3.3. Prediction of CpGs in the Promoter Regions of FHIT and PIAS1 Genes

**Figure 3:**
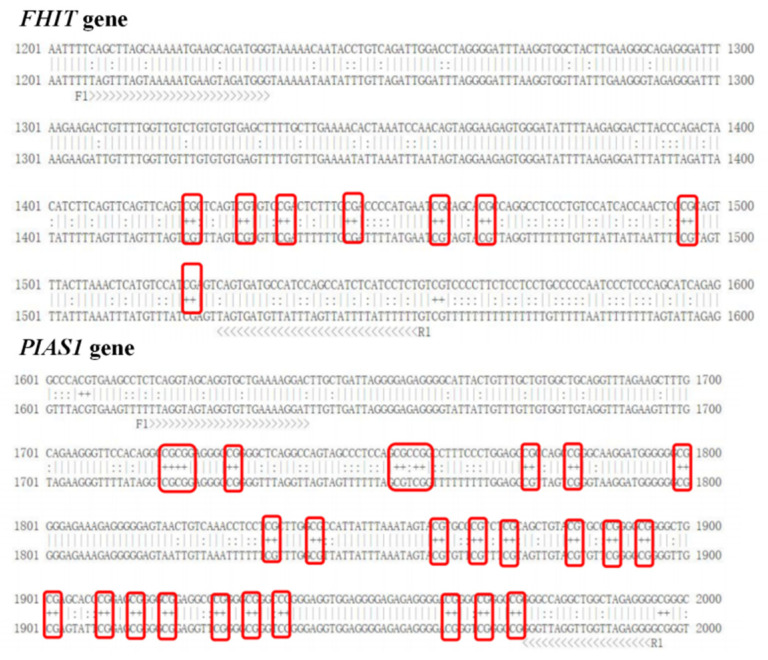
DNA methylation sites of *FHIT* and *PIAS1* genes in Xinjiang brown cattle.

The results of CpG prediction showed that the *FHIT* gene promoter region contained eight potential CpGs, and the promoter region of the *PIAS1* gene contained twenty-six potential CpGs (Figure 3).

### 3.4. Detection of Methylation Modifications in the Promoter Regions of FHIT and PIAS1 Genes

**Figure 4:**
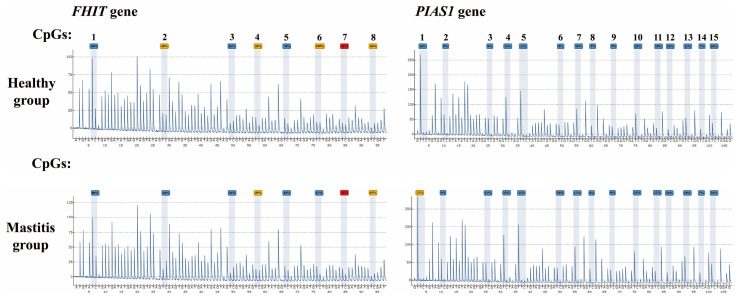
Methylation sequencing peaks of Xinjiang brown cattle mastitis *FHIT* and *PIAS1* genes in healthy and mastitis groups.

Pyrosequencing results showed that eight methylation sites were detected in the *FHIT* gene promoter and fifteen methylation sites were detected in the *PIAS1* gene promoter (Figure 4). In addition, the methylation level of the *FHIT* gene promoter region was 58.00 ± 23.52% in the healthy group and 65.97 ± 19.82% in the mastitis group, with no significant difference between the two groups (*p* > 0.05), and the overall methylation level was higher in the mastitis group. The methylation level in the *PIAS1* gene was 12.17 ± 4.25% in the healthy group and 11.48 ± 4.12% in the mastitis group, with no significant difference between the two groups (*p* > 0.05), and the overall methylation level was lower in the mastitis group.

The results further show that the methylation level of the CpG6 site of the *FHIT* gene promoter was higher in the healthy group, higher than that in the mastitis group, and the methylation levels of CpG3, CpG5, and CpG8 sites were significantly higher in the mastitis group (*p* < 0.01). In the promoter region of the *PIAS1* gene (Figure 4), the methylation levels of CpG2 and CpG12 sites were higher in the mastitis group than in the healthy group, the methylation levels of other CpG sites were lower in the mastitis group, and the methylation level of the CpG15 site was significantly lower in the mastitis group (*p* < 0.01).

### 3.5. Analysis of Relative Expression of Genes Related to Mastitis Resistance in Xinjiang Brown Cattle

**Figure 5:**
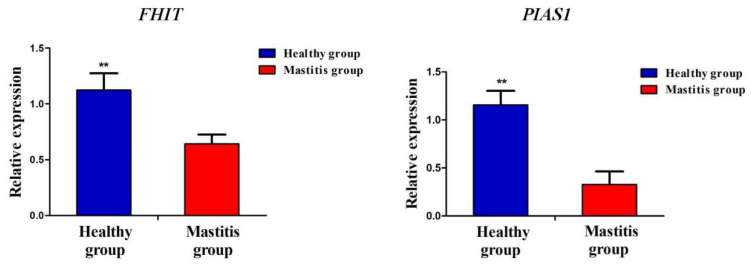
Analysis of relative expression levels of mastitis-resistance-related genes *FHIT* and *PIAS1* in healthy and mastitis cattle. ** represents *p* < 0.01.

The results of RT-qPCR showed that the *FHIT* and *PIAS1* genes were expressed in the blood of cattle in the healthy and mastitis groups, and the expression levels of both genes were significantly higher in blood from healthy cattle than those with the mastitis group (*p* < 0.01) (Figure 5).

### 3.6. Correlation Analysis between the Expression Levels of FHIT and PIAS1 Genes and Methylation Levels

Correlation analysis between gene expression and promoter methylation levels showed that there was no significant negative correlation between the methylation and expression levels of the *FHIT* gene promoter (*p* > 0.05, r^2^ = −0.185) and a significant positive correlation between the *PIAS1* gene expression and promoter methylation level (*p* < 0.05, r^2^ = 0.602).

## 4. Discussion

In this study, using GWAS, we found four SNPs associated with mastitis traits, which were located on chromosomes 2, 10, 18, and 29, and involved six genes. These six genes (*ZRANB3* [21,22], *PIAS1* [23,24,25], *ACTR3* [26,27], *LPCAT2* [28], *MGAT5* [29,30], and *SLC37A2* [31]) are all potentially associated with mastitis resistance (Table S5). Some studies have shown that, among them, the *PIAS1* gene is involved in the NF-kB and JAK/STAT signaling pathways, which are closely associated with mastitis [25,32]. The *PIAS1* gene interacts with transcription factors in these signaling pathways to regulate inflammatory cell adhesion and inhibit the process of inflammatory damage [25]. Liu et al. found that the *PIAS1* gene regulates breast tumorigenesis through selective epigenetic gene silencing [24]. In addition, *FHIT* is a well-known tumor suppressor gene and covalently binds to the cofactor intracellular diadenosine triphosphate (Ap3A). Some studies have shown that FHIT-Ap3A may inhibit tumor activity [33,34]. Ju et al. found that two indels in the *FHIT* gene significantly affected the milk somatic cell count in Xinjiang brown cattle [35]. Ju et al. also studied the correlation between eight indels in the *FHIT* gene and milk production traits in 388 Xinjiang brown cows, and found that P5-21bp was significantly associated with the milk somatic cell count of first- and sixth-parity cows (*p* < 0.05) [36]. Finally, we selected the *PIAS1* and *FHIT* genes and further investigated their effect on mastitis in terms of epigenetics.

Further analysis showed that the methylation levels of the *FHIT* and *PIAS1* genes were higher in the mastitis group than in the healthy group. Meanwhile, the expression of the *FHIT* gene was significantly higher in the blood from the healthy group compared to the mastitis group. Nasr et al. found that Egyptian breast cancer patients had higher methylation of the *FHIT* gene promoter than healthy people [37]. Syeed et al. studied the methylation of the *FHIT* gene promoter in 130 breast cancer patients using PCR-SSCP, DNA sequencing, and methylation-specific PCR. The results of that study showed that mutations in the *FHIT* gene were significantly associated with hypermethylation of the promoter region, resulting in complete inactivation of the *FHIT* gene and leading to breast cancer development [38]. Raish et al. studied the correlation between promoter methylation and expression of the *FHIT* gene in breast cancer patients in northern India and found that breast cancer tissue showed higher *FHIT* gene promoter methylation levels than the normal or adjacent tissue and a low expression of the *FHIT* gene was significantly related to promoter methylation [39]. Our results are similar to the results of these previous studies. The expression of the *FHIT* gene was reduced in Xinjiang brown cattle with mastitis, and gene expression levels were negatively correlated with methylation levels of the *FHIT* gene promoter. In a word, we consider that hypermethylation of the *FHIT* gene promoter region may lead to the suppression or reduction in *FHIT* gene expression, inhibiting its transcriptional activity and affecting the occurrence and development of mastitis.

In addition, Liu et al. found that *PIAS1* expression was elevated in breast tumor samples and knocking out the *PIAS1* gene in breast cancer cells could inhibit their growth in vivo [40]. However, in our study, the expression of the *PIAS1* gene was down-regulated in the mastitis group, and its expression was significantly positively correlated with the methylation level of the *PIAS1* gene promoter. This is inconsistent with previous studies and may be related to the different species. Hence, it is necessary to further study the expression of this gene at the protein level to determine its role in the molecular mechanisms of the development of mastitis. Finally, we consider that the methylation of the *FHIT* and *PIAS1* genes affects the expression of the genes to a certain extent and can be used as an epigenetic marker for mastitis resistance in Xinjiang brown cattle. In a word, only the candidate genes for mastitis resistance in Xinjiang brown cattle were examined at the genomic, epigenetic, and transcriptional levels in this study. The subsequent stage involves performing a thorough investigation and testing the *FHIT* and *PIAS1* genes at the protein and cellular levels. The identification of these candidate genes and associations between methylation of the promoter regions of candidate genes and resistance to mastitis provided a theoretical basis for improving the resistance of dairy cattle mastitis from the perspective of epigenetics.

## 5. Conclusions

In this study, six candidate genes associated with mastitis resistance were screened using GWAS. Changes in the promoter methylation levels of *FHIT* and *PIAS1* may influence the expression of these genes to further regulate the development of mastitis. The decrease in the methylation level in the promoter region of the *FHIT* gene may enhance resistance to mastitis in Xinjiang brown cattle. Our study provides a reference for research on mastitis resistance in Xinjiang brown cattle.

## Figures and Tables

**Table 1:** Genome-wide association analysis of significant SNPs and the nearest candidate genes of significant SNPs.

SNP Name	Chromosome	Position	*p*-Value	Candidate Genes	
				Name	Distance (kb)
BovineHD1800006960	18	22647995	2.91 × 10^−12^	*LPCAT2*	1246
BovineHD0200018606	2	64455540	1.32 × 10^−8^	*MGAT5*	1146
				*ZRANB3*	2172
				*ACTR3*	1433
Hapmap54158-rs29026721	29	28559588	2.04 × 10^−7^	*SLC37A2*	319
BovineHD1000004910	10	14760795	3.49 × 10^−7^	*PIAS1*	182

## Data Availability

The data that support the findings of this study are available from the corresponding author, X.H., upon reasonable request.

## Supplementary Materials

The following supporting information can be downloaded at: https://www.mdpi.com/article/10.3390/genes14061189/s1. Additional file S1: Table S1 Pyrosequencing primer sequences of *FHIT* and *PIAS1* genes. Additional file S2: Table S2 Reaction system of PCR (50 μL). Additional file S3: Table S3 The amplified conditions of PCR. Additional file S4: Table S4 The information of RT-qPCR primer. Additional file S5: Table S5 The functions of candidate genes.
