# Supplementary material for: Methylation Levels in the Promoter Region of FHIT and PIAS1 Genes Associated with Mastitis Resistance in Xinjiang Brown Cattle
Source: Genes (Basel). 2023 May 29;14(6):1189. doi: 10.3390/genes14061189 (PMC10298475; doi:10.3390/genes14061189)
Supplement: Supplementary file 1 [file genes-14-01189-s001.zip › genes-2334215-supplementary/Additional file S1.pdf]

Table S1 Pyrosequencing primer sequences of *FHIT* and *PIAS1* genes.

Gene	Primer sequence (5'→3')	Product size (bp)	Annealing temperature (℃)
FHIT	FH1T-F: TTGATGTTGGGAGGGATTGG<br>FH1T-R (Q48, 5'-Biotin): ACTTACCCAAACTACATCTTCAA<br>FH1T-S: ATGGTTGGATGGTATTAT	212	59.9
PIAS1	PIAS1-F:GGAGAAAGAGGGGGAGTAATTGTTAA<br>PIAS1-R (Q48, 5'-Biotin):TAAACCCCCCCCTCTAACCAACCT<br>PIAS1-2S: GGGGAGTAATTGTTAAATT	202	62.2
