# Supplementary material for: Methylation Levels in the Promoter Region of FHIT and PIAS1 Genes Associated with Mastitis Resistance in Xinjiang Brown Cattle
Source: Genes (Basel). 2023 May 29;14(6):1189. doi: 10.3390/genes14061189 (PMC10298475; doi:10.3390/genes14061189)
Supplement: Supplementary file 1 [file genes-14-01189-s001.zip › genes-2334215-supplementary/Additional file S2.pdf]

Table S2 Reaction system of PCR (50 μL).

**Component**	**Volume (μL)**
Forward primer (10 μM) (50pM/ul)	1.0
Reverse primer (10 μM) (50pM/ul)	1.0
The modified DNA	2.0
5×buffer GC (KAPA)	10.0
dNTP (10mM/each)	1.0
Taq enzyme (5U/μL)	0.2
dd H_2O	34.8
