# Supplementary material for: Methylation Levels in the Promoter Region of FHIT and PIAS1 Genes Associated with Mastitis Resistance in Xinjiang Brown Cattle
Source: Genes (Basel). 2023 May 29;14(6):1189. doi: 10.3390/genes14061189 (PMC10298475; doi:10.3390/genes14061189)
Supplement: Supplementary file 1 [file genes-14-01189-s001.zip › genes-2334215-supplementary/Additional file S3.pdf]

Table S3 The amplifided conditions of PCR.

Temperature	Time	Cycle
95 ℃	3 min	1
94 ℃	30 s	
56℃	30 s	40
72 ℃	1 min	
72 ℃	7 min	1
4 ℃	Forever	1
