# Supplementary material for: Methylation Levels in the Promoter Region of FHIT and PIAS1 Genes Associated with Mastitis Resistance in Xinjiang Brown Cattle
Source: Genes (Basel). 2023 May 29;14(6):1189. doi: 10.3390/genes14061189 (PMC10298475; doi:10.3390/genes14061189)
Supplement: Supplementary file 1 [file genes-14-01189-s001.zip › genes-2334215-supplementary/Additional file S4.pdf]

Table S4 The information of RT-qPCR primer.

Genes	Primer Sequencing	Produce length/bp	Annealing temperature (℃)
FHIT	F:AACTGTCCTTTGCCCTGGTGAAC<br>R:CGACTCTCTGTGCTGCCTGAAAC	138	61
PIAS1	F:GCCATCTGCCAAGAGAACCTGTC<br>R:ACTGGAGATGCTTGATGTGGAAGAC	93	60
GAPDH	F:GAGATCAAGAAGGTGGTGAAGCAG<br>R:GTAGAAGAGTGAGTGTCGCTGTTG	102	58
