# Supplementary material for: Methylation Levels in the Promoter Region of FHIT and PIAS1 Genes Associated with Mastitis Resistance in Xinjiang Brown Cattle
Source: Genes (Basel). 2023 May 29;14(6):1189. doi: 10.3390/genes14061189 (PMC10298475; doi:10.3390/genes14061189)
Supplement: Supplementary file 1 [file genes-14-01189-s001.zip › genes-2334215-supplementary/Additional file S5.pdf]

Table S5 The functions of candidate genes.

Candidate genes	Gene name	function
LPCAT2	lysophosphatidylcholine acyltransferase 2	It is involved in the synthesis of platelet activating factors, the production of biofilms in inflammatory cells, the synthesis of glycerol phospholipid precursors, the main components of cell membranes, key enzymes that regulate phospholipid metabolism, and the formation of lipid droplets
MGAT5	mannosyl (alpha-1,6-)-glycoprotein beta-1,6-N-acetyl-glucosaminyltransferase	One of the most important enzymes involved in the activation of cell signaling pathways, regulation of glycoprotein-oligosaccharide biosynthesis, recombination of actin cytoskeleton, cell adhesion and cell migration
ZRANB3	Zinc finger RANBP2-type containing 3	Involved in endonuclease activity and nucleic acid binding
ACTR3	ARP3 actin related protein 3 homolog	Regulates inflammation, participates in the regulation of actin polymerization, and is a major component of the cytoskeleton
SLC37A2	solute carrier family 37 member 2	Regulate the activity of transporters
PIAS1	protein inhibitor of activated STAT 1	It is involved in many cellular processes and plays a central role in transcriptional regulation of JAK-STST and NF-kB signaling pathways
FHIT	fragile histidine triad	Regulates the expression of genes critical for cell proliferation and survival, regulates SRC and AKT1 signaling pathways and induces apoptosis and tumor suppressor factors
DYRK2	dual specificity tyrosine phosphorylation regulated kinase 2	Regulates the expression of genes critical for cell proliferation and survival, regulates SRC and AKT1 signaling pathways and induces apoptosis and tumor suppressor factors
